# Trajectories of mortality risk among patients with cancer and associated end-of-life utilization

**DOI:** 10.1038/s41746-021-00477-6

**Published:** 2021-07-01

**Authors:** Ravi B. Parikh, Manqing Liu, Eric Li, Runze Li, Jinbo Chen

**Affiliations:** 1 Department of Medical Ethics and Health Policy, Perelman School of Medicine, University of Pennsylvania, Philadelphia, PA USA; 2 Department of Medicine, Perelman School of Medicine, University of Pennsylvania, Philadelphia, PA USA; 3 Abramson Cancer Center, University of Pennsylvania, Philadelphia, PA USA; 4 Leonard Davis Institute of Health Economics, University of Pennsylvania, Philadelphia, PA USA; 5 Department of Statistics, Penn State University, University Park, PA USA; 6 Department of Biostatistics, Epidemiology, and Informatics, Perelman School of Medicine, University of Pennsylvania, Philadelphia, PA USA

**Keywords:** Prognosis, Health policy, Palliative care, Cancer

## Abstract

Machine learning algorithms may address prognostic inaccuracy among clinicians by identifying patients at risk of short-term mortality and facilitating earlier discussions about hospice enrollment, discontinuation of therapy, or other management decisions. In the present study, we used prospective predictions from a real-time machine learning prognostic algorithm to identify two trajectories of all-cause mortality risk for decedents with cancer. We show that patients with an unpredictable trajectory, where mortality risk rises only close to death, are significantly less likely to receive guideline-based end-of-life care and may not benefit from the integration of prognostic algorithms in practice.

High-quality end-of-life (EOL) care is a national priority for patients with serious illness^1^, particularly during the coronavirus disease 2019 pandemic as these patients are at higher risk for mortality than the general population^2^. Prognostic inaccuracy among clinicians contributes to low-quality EOL care, including delayed hospice utilization and increased acute care utilization close to death, for patients with serious illnesses, such as cancer^3,4^. Machine learning (ML) algorithms outperform traditional tools used for prognostication and may facilitate earlier clinician–patient discussions about hospice enrollment, discontinuation of therapy, or other management decisions^5^. Most research to date has reported on the performance of static predictions of mortality risk from ML algorithms^5–7^. However, patients’ risk of mortality may change over time in non-linear patterns. Identifying trajectories of mortality risk may help inform how clinicians and health systems can implement prognostic algorithms, including understanding which populations such algorithms are likely to benefit. To that end, we identified trajectories of all-cause mortality risk and their association with existing metrics of EOL care quality, using a longitudinal prospective cohort of patients with cancer.

To calculate mortality risk, we used a previously trained gradient boosting machine (GBM), an ensemble ML algorithm, based on 559 structured electronic health record (EHR) variables for 26,525 patients with cancer who were seen at 11 academic or community medical oncology practices within a large academic cancer center in 2016^8^. The training and prospective validation of this algorithm among a more recent cohort seen at 18 oncology practices have been previously published^5,8^. This algorithm was integrated into our EHR in 2018. Every Thursday morning, the algorithm generated predictions of all-cause mortality risk for all patients with a scheduled encounter during the following week with an eligible clinician. In the prospective validation, the *c*-statistic of our GBM across all disease cohorts was 0.89 (95% confidence interval [CI] 0.88–0.90), ranging from 0.74 to 0.96 across difference disease groups, and the sensitivity was 67.2% at a 10% threshold of mortality risk^5^. To identify distinct trajectories of mortality risk among decedents, we identified 3280 individuals who died of any cause between January 2, 2018 and May 4, 2020, who had at least 3 face-to-face visits in an oncology practice for a cancer diagnosis in the year preceding death, and who had at least 2 visits in the 6 months prior to death. The purpose of the latter two inclusion criteria was to capture a cohort comprised of primary patients within the oncology practice who would have EHR data from which to generate predictions. We focus on decedents (individuals who die) because these individuals have associated EOL care quality metrics.

Of those 3280 individuals, the median number of encounters in the 6 months prior to death was 6 (interquartile range 3–10) (Supplementary Table 1). Each of those encounters had an associated prospective mortality risk prediction from the GBM. We used functional principal component analysis (FPCA)—a statistical method for identifying modes of variation via calculating functional principle component (FPC) scores and eigenfunctions for time-varying data—to all mortality risk predictions associated with encounters in the 6 months prior to death. The main advantages of FPCA over other methods are that FPCA, as a nonparametric method, does not assume normality on the distribution of the data and that this algorithm takes both mean and covariance functions into account (see “Methods,” Eqs. 1–6)^9–12^.

FPCA revealed 2 dominant modes of variation that explained over 95% of variation (Fig. 1). The first FPC explained 84.1% of all variation, and the second FPC explained 11.5% of all variation. Clusters of patients were derived using an expectation–maximization (EM) algorithm^13,14^ based on the FPC scores obtained. The first cluster represented 36.1% of all patients in the cohort. This group (heretofore referred to as “unpredictable”) consisted of patients whose average trajectory was characterized by a low risk of mortality from 6 months until approximately 30 days prior to death, after which mortality risk rose sharply. The second cluster represented 63.9% of all patients in the cohort. This group (heretofore referred to as “predictable”) consisted of patients whose average trajectory was characterized by a relatively higher baseline risk of mortality that constantly rose until death. The overall shape of these trajectories was similar when using encounter data up to 1 year prior to death (range of encounters 2–51; see Supplementary Fig. 1). We conducted a sensitivity analysis to assess the robustness of our method by splitting the decedent cohort into training (75%) and validation (25%) subcohorts, running the FPCA algorithm on the training cohort and projecting the validation cohort onto the resulting FPC scores for each patient. We found that the projected FPC scores overlapped with the FPC scores from the training data in similar areas defined by the two FPC coordinates, suggesting the robustness of our method.

**Fig. 1:**
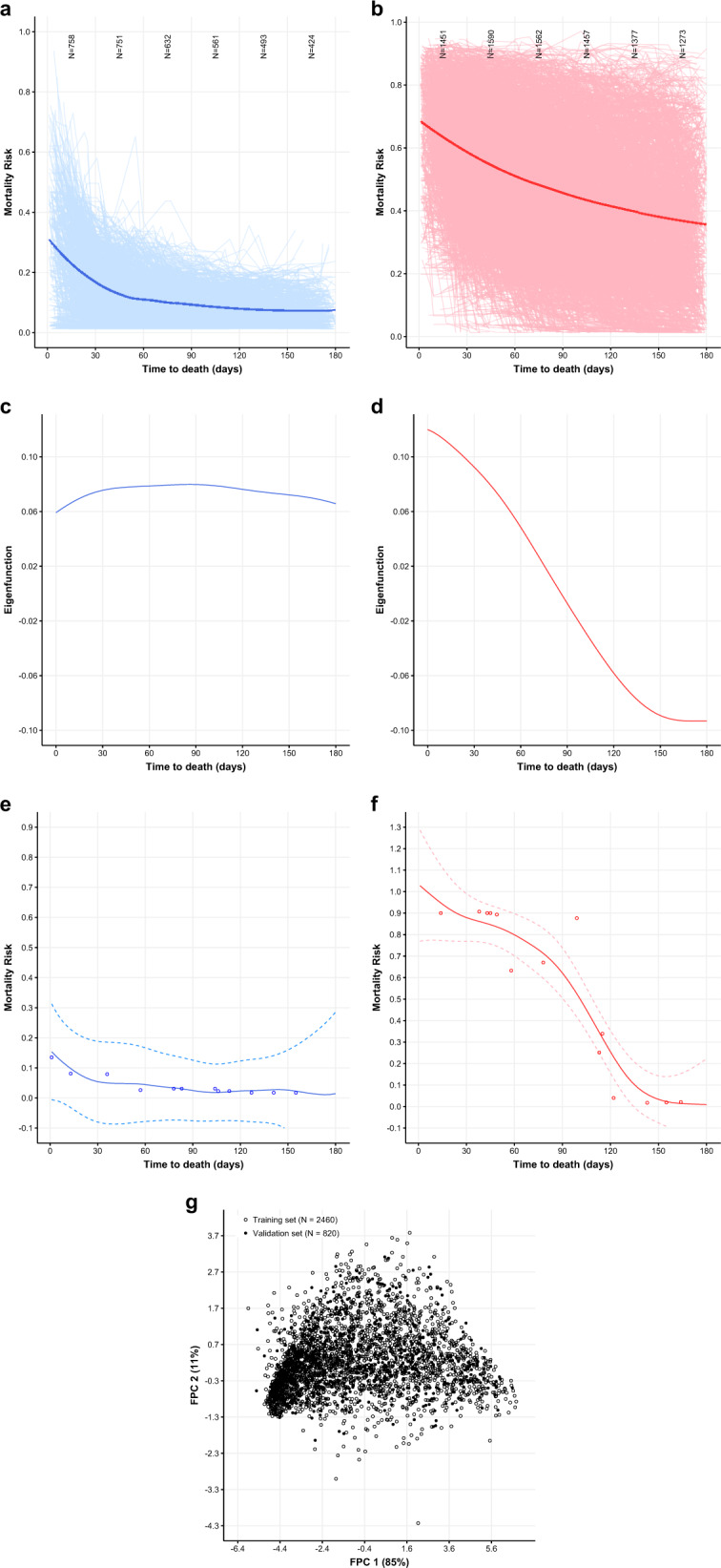
Dominant modes of variation of trajectories of mortality risk. **a** First (unpredictable) trajectory derived from FPCA. Smoothed estimate of the mean function for mortality risk scores from local weighted regression (loess) method (blue smoothed line), superimposed on the individual trajectories for all patients in this FPC (blue spaghetti plot). **b** Second (predictable) trajectory derived from FPCA. Smoothed estimate of the mean function for mortality risk scores from local weighted regression (loess) method (red smoothed line), superimposed on the individual trajectories for all patients in this FPC (red spaghetti plot). **c** Smoothed estimates of the first eigenfunction from FPCA (blue line), representing the first mode of variation from the “unpredictable” trajectory that explains 84.1% of total variation. **d** Smoothed estimates of the second eigenfunction from FPCA (red line), representing the second mode of variation from the “predictable” trajectory that explains 11.5% of total variation. **e** The individual patient with largest absolute value of the projection on the first eigenfunction (among the unpredictable trajectory) who had ≥10 encounters. Mortality risks (blue circles), predicted trajectories (blue solid lines), 95% simultaneous bands (dashed blue lines). **f** The individual patient with the largest absolute value of projection on the second eigenfunction (among the predictable trajectory) who had ≥10 encounters. Mortality risks (red circles), predicted trajectories (red solid lines), 95% simultaneous bands (dashed red lines). **g** Plotting FPC 1 (*x* axis) against FPC 2 (*y* axis) for the training set (empty circles) and validation set (filled circles). Both pairs of FPCs explain above 95% of the total variation in each set.

We used internal cancer registry and EHR data to characterize each mortality risk trajectory (see Supplementary Table 1). Individuals in the “predictable” mortality risk trajectory were more likely to be married and have more clinical encounters near death, high comorbidity burden, worse performance status, stage IV disease, and gastrointestinal malignancies. Individuals in the “unpredictable” mortality risk trajectory were more likely to have hematologic and primary central nervous system (CNS) malignancies.

To identify the association between mortality risk trajectory and EOL outcomes, we studied 4 common metrics of high-quality EOL care: hospice enrollment prior to death, dying outside of the hospital, no intensive care unit (ICU) admission in the last 30 days of life, and no chemotherapy in the last 14 days of life^1^. Adherence to each of these constitutes high-quality EOL care. We found that, compared to unpredictable trajectories, predictable mortality trajectories were associated with higher hospice enrollment (adjusted odds ratio [aOR] 1.87, 95% CI 1.48–2.37), less inpatient death (aOR 0.72, 95% CI 0.56–0.92), less EOL ICU admissions (aOR 0.74, 95% CI 0.57–0.95), and less chemotherapy near the end of life (aOR 0.77, 95% CI 0.55–1.08) (Fig. 2). Supplementary Fig. 2 describes observed rates of guideline-based EOL care between the predictable and unpredictable trajectories.

**Fig. 2:**
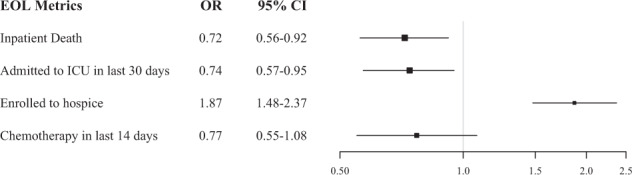
End-of-life outcomes. Logistic regression where the predictors are baseline patient characteristics plus mortality risk trajectories (Predictable vs. Unpredictable), and the outcome is each of the end-of-life (EOL) metric. An odds ratio >1 indicates that patients assigned to the “Predictable” trajectory are more likely to have the corresponding EOL care compared to those assigned to the “Unpredictable” trajectory.

We report that a large percentage of deaths from cancer (36.1%) follow an unpredictable disease trajectory, defined as a rise in mortality risk very close to death as opposed to a consistent rise in mortality risk long before death. Patients with unpredictable mortality trajectories prior to death have fewer clinical encounters close to death, better performance status, lower comorbidity burden, and lower rates of hematologic and CNS malignancies compared to patients with predictable mortality trajectories. Lower baseline utilization, and subsequently lower rates of laboratory and comorbidity assessment, could contribute to patients following an unpredictable mortality risk trajectory. However, even patients with relatively high utilization were present in the unpredictable risk trajectory (see Fig. 1e), suggesting that certain adverse laboratory values and comorbidities may not manifest until close to death among those with unpredictable trajectories. Many common EOL metrics, particularly hospice enrollment, are predicated on reliable estimation of 6-month mortality risk. Furthermore, earlier enrollment in hospice may facilitate more goal-concordant care, better symptom management, and avoidance of acute care prior to death^1^. While providing estimates of mortality risk may improve clinicians’ awareness of short-term mortality risk and facilitate higher-quality EOL care, this strategy is only likely to be successful for individuals with predictable mortality trajectories. For individuals with unpredictable disease trajectories, overreliance on risk predictions from prognostic algorithms to guide care could delay important EOL care and reinforce pre-existing inequities in outcomes such as hospice referral and aggressive treatment near the end of life. Subgroups of individuals with unpredictable mortality trajectories may instead benefit from early concurrent palliative care and/or hospice, close to the original diagnosis of serious illness and not necessarily triggered by an algorithm, to ensure adequate symptom control and advance care planning prior to death.

There were limitations to the study. We lacked EOL quality data on 11.6% of the decedent cohort, who were excluded from the analysis. Second, our algorithm relied on EHR data, which has good performance in predicting mortality but may not capture patient-centric data or physician-specific factors that could improve the sensitivity of the prediction. Third, while we identified trajectories of mortality risk, any findings regarding absolute levels of mortality risk within trajectories must be interpreted with caution given that the algorithm had suboptimal calibration for mortality risks ≥40%. Fourth, we focus on four common EOL measures. While there are other metrics of EOL quality, these are among the most commonly used in oncology quality reporting guidelines. Fifth, we assumed that differential encounter data and frequency were reflective of differences in clinician-assessed severity of illness rather than other potential sources of missingness that was not at random. Given that this is a cancer cohort among individuals who received their primary oncologic care in our center, it is unlikely that there was significant missingness in encounter-level data. Sixth, we only used data within a single academic health system and thus results may not be generalizable to other oncology care settings, although we used data from a variety of practices with a good mix of community and tertiary academic oncology practices. Finally, further work clustering the entire cohort of non-decedents and decedents is necessary to inform how clinicians could incorporate these trajectories in their clinical assessment. Preliminary data (Supplementary Fig. 3) suggest that our FPCA algorithm is able to adequately distinguish between distinct trajectories among non-decedents.

## Methods

### Data sources

The study cohort was extracted from Clarity, a database that contains structured data elements of individual EHR data for patients treated at the University of Pennsylvania Health System (UPHS). The EHRs contained patient demographic characteristics, comorbidities, laboratory results, and utilization data. We linked individual patient EHRs from Clarity to the University of Pennsylvania Oncology Registry (UPOR) to obtain cancer type and stage when determining the characteristics of patients in each trajectory group. Mortality data were derived from internal administrative data, the EHR, and the Social Security Administration Death Master File, matched to UPHS patients by social security number and date of birth^15^. EOL outcomes were derived from the Abramson Cancer Center cancer registry, EHR data, and an external agency used at UPHS to capture dates of death from local obituary data. EOL outcome data was primarily available for individuals with three face-to-face encounters in the year prior to death. EOL hospitalizations encompassed both oncologic and non-oncologic hospitalizations and were only able to be ascertained from UPHS inpatient stays.

### Ethics

The University of Pennsylvania Institutional Review Board approved this study with a waiver of informed consent, classifying this study as quality improvement.

### Participants

Patients were eligible if they were 18 years or older and had at least two encounters at one of the 18 medical oncology clinics within the UPHS between January 2, 2018 and May 4, 2020. To ensure that we captured patients who received their primary oncology care in our center and health system, we only included patients who had at least three face-to-face visits in an oncology practice for a cancer diagnosis in the year preceding death and at least two visits in the 6 months prior to death. Of the 44,588 patients who met the criteria above, 41,308 (92.6%) patients who were alive at 6 months from the index encounter were excluded from this analysis. Three thousand two hundred and eighty deceased patients who had encounters within 6 months of death were eligible for this study.

### Predictive algorithm

The mortality risks of patients were derived from a GBM learning algorithm designed to predict 180-day mortality among outpatients with cancer. Five hundred and fifty-nine structured EHR features collected at UPHS were used to train this algorithm. The 180-day prospective predictions were generated once a week on Thursdays. Detailed descriptions of the ML algorithm are described in previous publications^5,8^. The overall AUC of this algorithm was 0.89 (95% CI, 0.88–0.90), and disease-specific AUC ranged from 0.74 to 0.96^5^.

### Outcomes

We were interested in determining the association between identified trajectories of mortality risks and four EOL outcomes: inpatient death, admission to ICU in the last 30 days of life, hospice enrollment prior to death, and receipt of chemotherapy in the last 14 days of life.

### Features

To determine distinct trajectory patterns during the end of life, we used predicted mortality risks from the GBM for each patient encounter that occurred within 180 days prior to death. To characterize patients after we identified distinct trajectory groups, we used baseline patient characteristics collected from the Clarity and UPOR data. Variables used for characterization are provided in Supplementary Table 1.

### Statistical analysis

The distinct patterns of trajectories of mortality risks were ascertained using FPCA. The rationale for choosing this method instead of other clustering techniques, such as group-based trajectory modelling and *K*-means, are threefold: (1) Time points when the predicted risks were generated differed from patient to patient. Since patients went to clinics based on their own preferences, the intervals between each visit vary; (2) It is almost impossible to assume an overall distribution of the shape of mortality risks. The trajectory for each patient vary between flattened straight lines (if a patient only had two or three predictions) and U-shapes; and (3) Given the variation of shapes of each patient’s risk trajectory, it is important to consider both mean functions and covariance structures during the clustering process. FPCA can account for all three of these features of longitudinal GBM-predicted mortality risks, while the other two methods (group-based trajectory modelling and *K*-means) cannot^16,17^.

We used the fdapace R package in CRAN to perform the FPCA analysis that considered the sparsity of the data^9–12^. Let *Y*_*ij*_ denote the mortality risk of the *i*th patient observed from trajectory *X*_*i*_(*t*) at the *j*th time point *T*_*ij*_ where *T*_*ij*_ is irregular. Then *Y*_*ij*_ can be modeled as Eq. (1)

1 $$Y_{ij} = X_i\left( {T_{ij}} \right) + \varepsilon _{ij},$$

where $\varepsilon _{ij}$ denotes random error; *i* = 1,…,*n*; *j* = 1,…,*N*_*i*_. The number of predicted risks *N*_*i*_ for the *i*th subject was small and considered random. The major steps are summarized as follows:

The smoothed mean $\hat \mu$ was estimated using local linear smoothing that aggregates all available mortality risks together. Raw covariance for each curve was calculated as Eq. (2).

2 $$G_i\left( {T_{ij},T_{il}} \right) = \left( {Y_{ij} - \hat \mu \left( {T_{ij}} \right)} \right)\left( {Y_{il} - \hat \mu \left( {T_{il}} \right)} \right),$$

then all of the raw covariances were aggregated to generate the sample raw covariance. The smoothed covariance $\hat G\left( {s,t} \right)$ was then estimated using the off-diagonal elements of the sample raw covariance. The estimated *k*th eigenfunction $\hat \varphi _k$ and eigenvalue $\hat \lambda _k$ were calculated via eigenanalysis on the smoothed covariance by solving the integral Eq. (3).

3 $${\int}_T {\hat G\left( {s,t} \right)\hat \varphi _k\left( s \right){\rm{d}}s = \hat \lambda _k} \hat \varphi _k\left( t \right) \cdot$$

The “Functional Principal Components Analysis Through Conditional Expectation (PACE)” method was used to estimate the corresponding FPC scores in Eq. (4).

4 $$\hat \xi _{ik} = \hat E\left[ {\hat \xi _{ik}{\mathrm{|}}Y_i} \right] = \hat \lambda _k\hat \varphi _{ik}^T\mathop {\sum}\nolimits_{Y_i}^{ - 1} {(Y_i - \hat \mu _i).}$$

The number of FPC scores *K* sufficient to describe the shape of the predicted risks was determined by calculating the fraction of variance explained (FVE), and qualitative features of the longitudinal patterns of mortality risks were summarized by the corresponding eigenfunctions $\widehat \varphi _k$.

The trajectory *X*_*i*_(*t*) for the *i*th patient using the first *K* eigenfunctions was projected as Eq. (5).

5 $$\hat X_i^K\left( t \right) \equiv \hat \mu \left( t \right) + \mathop {\sum}\nolimits_{k = 1}^K {\hat \xi _{ik}\hat \varphi _k(t)} .$$

The 95% simultaneous confidence bands for *X*_*i*_(*t*) was constructed as Eq. (6).

6 $$\hat X_i^K\left( t \right) \pm \sqrt {\chi _{K,95{\mathrm{\% }}}^2\hat \varphi _{K,t}^T\hat {\Omega}_K\hat \varphi _{K,t}} ,$$

where $\chi _{K,95\% }^2$ was the 95th quantile of the Chi-squared distribution with *K* degrees of freedom, and $\hat {\Omega}_K$ was the estimated variance of $\hat \xi _{ik}$.

We identified two FPCs with 95% FVE, where the first FPC contributed 84.1% and the second 11.5%. We chose a cutoff of 95% since an FVE >90% is generally accepted as appropriately sensitive for identifying clinically relevant trajectories^18^. We assigned patients into two clusters based on the FPC scores using an EM algorithm for model-based clustering^13,14^. The mean trajectories of the two clusters were either “predictable” or “unpredictable”. To compare patients in the two clusters, we conducted logistic regression analysis to assess the association between the trajectory and patient characteristics, including baseline variables and variables related to cancer treatment.

To evaluate the association between trajectory and EOL outcomes, we fit separate logistic regression models using each of the four EOL metrics as dichotomous outcomes. The independent variable of interest was the trajectory (predictable vs. unpredictable), with all features in Supplementary Table 1 used as covariates. For all logistic regression analyses, missing indicators were added to address the issue of missing data, except for Eastern Cooperative Oncology Group performance status (ECOG) values, which were imputed via multiple imputations that repeated 50 times^19,20^.

### Reporting summary

Further information on research design is available in the Nature Research Reporting Summary linked to this article.

## Supplementary information

Supplementary Information

Reporting Summary

## Data Availability

The data shown in the manuscript are available upon request from the corresponding author.

## Code availability

All statistical analysis was performed in R version 3.6.0. The analysis codes can be obtained from https://github.com/ManqingLiu/Trajectory.Modelling.git. The core algorithm for FPCA can be obtained from https://github.com/functionaldata/tPACE. The validation for the GBM algorithm has been previous published (https://jamanetwork.com/journals/jamaoncology/article-abstract/2770698) and source code is available at https://github.com/pennsignals/eol-onc.
