# Supplementary material for: Trajectories of mortality risk among patients with cancer and associated end-of-life utilization
Source: NPJ Digit Med. 2021 Jul 1;4:104. doi: 10.1038/s41746-021-00477-6 (PMC8249647; doi:10.1038/s41746-021-00477-6)
Supplement: Supplementary file 1 — Supplementary Information [file 41746_2021_477_MOESM1_ESM.pdf]

SUPPLEMENT

Supplementary Table 1. Characteristics of mortality trajectories from logistic regression	2
Supplementary Figure 1. Trajectories and clustering of patients up to one year prior to death.	4
Supplementary Figure 2. Observed end-of-life outcomes by trajectory.	5
Supplementary Figure 3. Trajectory modelling among non-decedents, split by distinct trajectories (A and B).	6

Supplementary Table 1. Characteristics of mortality trajectories from logistic regression*

**Characteristics**	**OR†**	**95% CI**	**P value**
(Intercept)	0.14	[0.08-0.23]	<0.001
**Age**			
< 75 (Ref)			
≥ 75	0.73	[0.60-0.89]	0.002
**# of encounters**	1.22	[1.19-1.24]	<0.001
Year of death			
2018 (Ref)			
2019	0.87	[0.73-1.04]	0.133
2020	1.04	[0.77-1.39]	0.800
Gender			
Male (Ref)			
Female	0.97	[0.86-1.11]	0.689
Race			
Black (Ref)			
White	1.20	[0.95-1.53]	0.132
Other	1.19	[0.80-1.76]	0.397
Missing	1.17	[0.68-2.01]	0.560
**Marital status**			
Married (Ref)			
Unmarried	0.83	[0.69-1.00]	0.048
Insurance			
Commercial insurance (Ref)			
Managed Care	1.00	[0.70-1.43]	0.992
Medicaid	1.16	[0.70-1.91]	0.563
Medicare	0.93	[0.66-1.30]	0.654
Missing	0.58	[0.30-1.09]	0.089
**Elixhauser comorbidity score**			
0-1 (Ref)			
2	1.50	[1.16-1.95]	0.002
3+	3.69	[2.92-4.66]	<0.001
**ECOG performance status**			
0-1 (Ref)			
2+	1.31	[1.04-1.64]	0.020
**Cancer stage**			
Not IV (Ref)			
IV	2.02	[1.66-2.47]	<0.001
Hospital type			

General oncology (Ref)			
Tertiary academic	1.16	[0.94-1.43]	0.169
Cancer type			
Thoracic (Ref)			
Breast	1.15	[0.77-1.70]	0.494
**Gastrointestinal tract**	1.79	[1.37-2.33]	< 0.001
Genitourinary	1.35	[0.93-1.96]	0.118
Gynecology	1.57	[0.75-3.29]	0.235
Leukemia/Other Hematologic Malignancy	1.26	[0.82-1.96]	0.293
Lymphoma	0.72	[0.50-1.06]	0.094
Melanoma	0.84	[0.55-1.30]	0.435
**Myeloma**	0.62	[0.41-0.92]	0.019
**Neuro-oncology**	0.32	[0.21-0.49]	< 0.001
Other	0.58	[0.25-1.33]	0.197
Missing	0.90	[0.66-1.23]	0.504

* Bolded variables have statistically significant ORs.

†OR: “Predictable” trajectory vs. “Unpredictable” trajectory

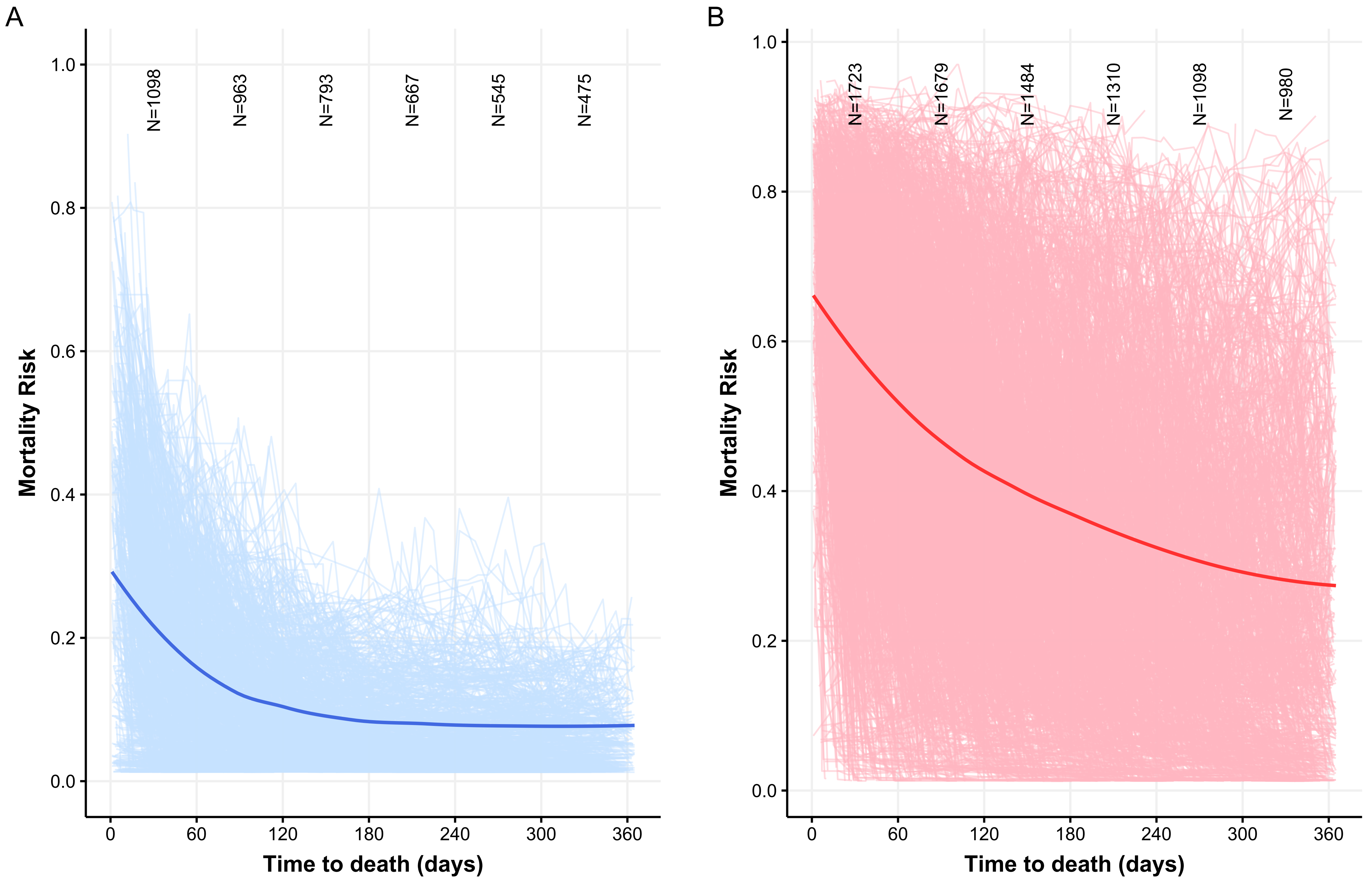

A
B
Mortality Risk
Time to death (days)
N=1098
N=963
N=793
N=667
N=545
N=475
N=1723
N=1679
N=1484
N=1310
N=1098
N=980
0.0
0.2
0.4
0.6
0.8
1.0
0
60
120
180
240
300
360

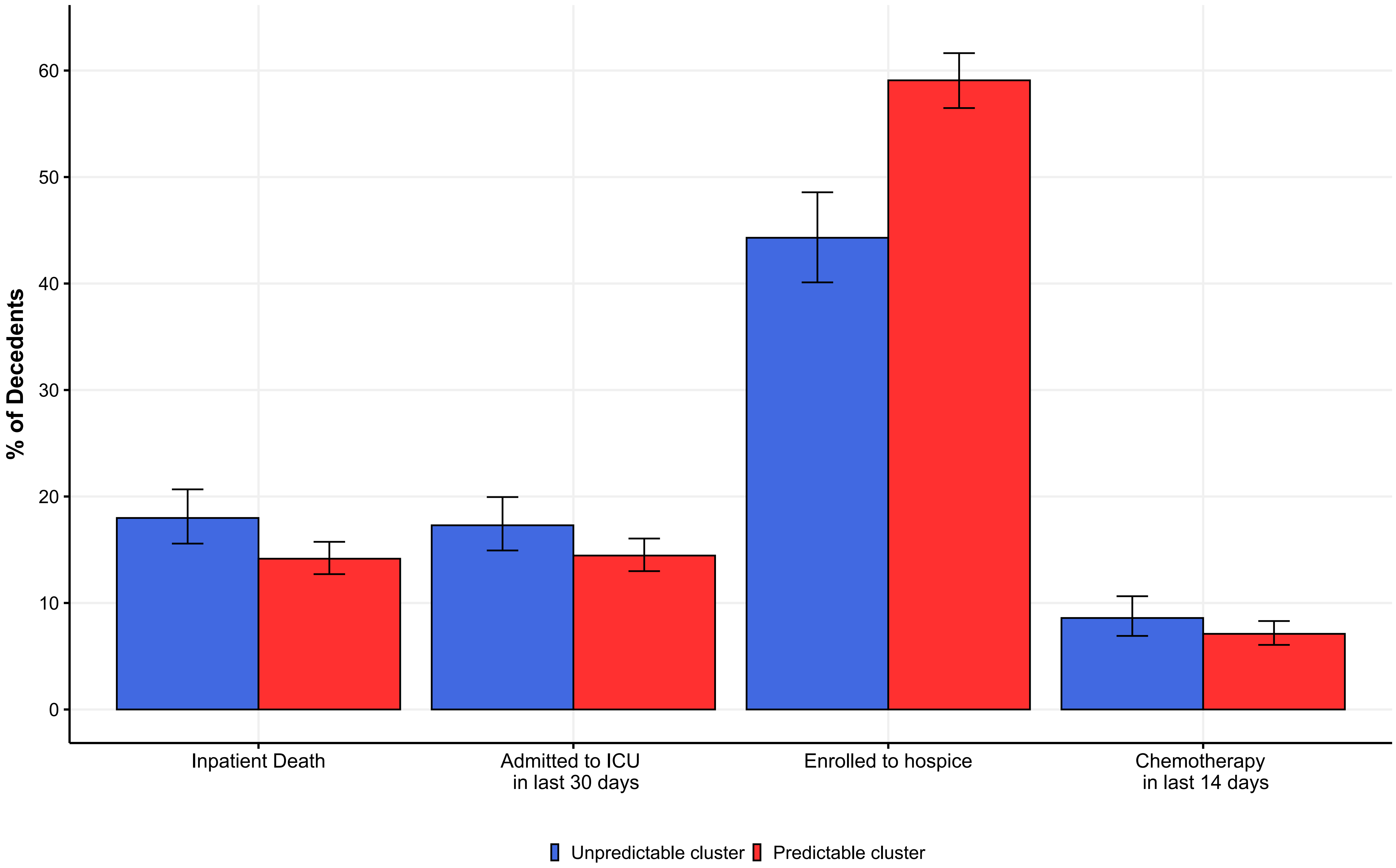
% of Decedents
0
10
20
30
40
50
60
Inpatient Death
Admitted to ICU
in last 30 days
Enrolled to hospice
Chemotherapy
in last 14 days
Unpredictable cluster
Predictable cluster

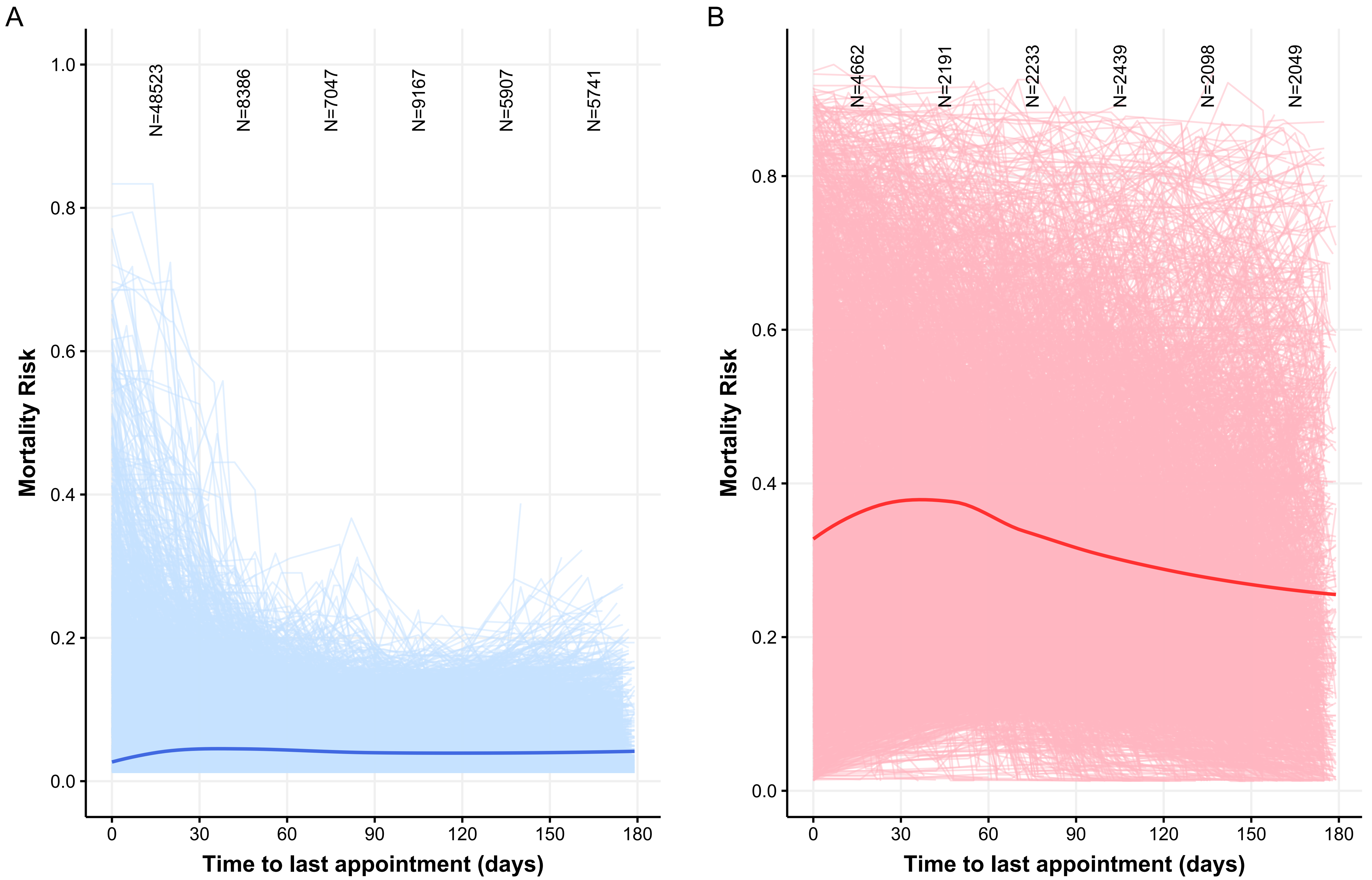
A
B
N=48523
N=8386
N=7047
N=9167
N=5907
N=5741
N=4662
N=2191
N=2233
N=2439
N=2098
N=2049
Mortality Risk
Time to last appointment (days)
0.0
0.2
0.4
0.6
0.8
1.0
0
30
60
90
120
150
180

Supplementary Figure 1. Trajectories and clustering of patients up to one year prior to death. A, First (“Unpredictable”) trajectory derived from EM algorithm using the elements from FPCA. Smooth Estimate of the Mean Function from Local weighted regression (loess) method (blue smoothed line); individual trajectory for all patients in this FPC (blue spaghetti plot). B, Second (“Predictable”) trajectory derived from EM algorithm using the elements from FPCA. Smooth Estimate of the Mean Function from Local weighted regression (loess) method (red smoothed line); individual trajectory for all patients in this FPC (red spaghetti plot).

Supplementary Figure 2. Observed end-of-life outcomes by trajectory. Observed rates of end-of-life outcomes, split by mortality trajectory (predictable [red] vs. unpredictable [blue]).

Supplementary Figure 3. Trajectory modelling among non-decedents, split by distinct trajectories (A and B).
