# Supplementary material for: Trajectories of mortality risk among patients with cancer and associated end-of-life utilization
Source: NPJ Digit Med. 2021 Jul 1;4:104. doi: 10.1038/s41746-021-00477-6 (PMC8249647; doi:10.1038/s41746-021-00477-6)
Supplement: Supplementary file 2 — Reporting Summary [file 41746_2021_477_MOESM2_ESM.pdf]

nature research

Corresponding author(s): Ravi B. Parikh MD MPP

Last updated by author(s): Apr 19, 2021

Reporting Summary

Nature Research wishes to improve the reproducibility of the work that we publish. This form provides structure for consistency and transparency in reporting. For further information on Nature Research policies, see our Editorial Policies and the Editorial Policy Checklist.

Statistics

For all statistical analyses, confirm that the following items are present in the figure legend, table legend, main text, or Methods section.

n/a	Confirmed	
☐	☒	The exact sample size (n) for each experimental group/condition, given as a discrete number and unit of measurement
☐	☒	A statement on whether measurements were taken from distinct samples or whether the same sample was measured repeatedly
☐	☒	The statistical test(s) used AND whether they are one- or two-sided<br>*Only common tests should be described solely by name; describe more complex techniques in the Methods section.*
☐	☒	A description of all covariates tested
☐	☒	A description of any assumptions or corrections, such as tests of normality and adjustment for multiple comparisons
☐	☒	A full description of the statistical parameters including central tendency (e.g. means) or other basic estimates (e.g. regression coefficient) AND variation (e.g. standard deviation) or associated estimates of uncertainty (e.g. confidence intervals)
☐	☒	For null hypothesis testing, the test statistic (e.g. F, t, r) with confidence intervals, effect sizes, degrees of freedom and P value noted<br>*Give P values as exact values whenever suitable.*
☒	☐	For Bayesian analysis, information on the choice of priors and Markov chain Monte Carlo settings
☐	☒	For hierarchical and complex designs, identification of the appropriate level for tests and full reporting of outcomes
☒	☐	Estimates of effect sizes (e.g. Cohen's d, Pearson's r), indicating how they were calculated

Our web collection on statistics for biologists contains articles on many of the points above.

Software and code

Policy information about availability of computer code

Data collection	The validation for the GBM algorithm has been previous published (https://jamanetwork.com/journals/jamaoncology/article-abstract/2770698) and source code is available at https://github.com/pennsignals/eol-onc .
Data analysis	The data shown in the manuscript are available upon request from the corresponding author.<br><br>All statistical analysis was performed in R version 3.6.0. The analysis codes can be obtained from https://github.com/ManqingLiu/Trajectory.Modelling.git.<br><br>The core algorithm for FPCA can be obtained from https://github.com/functionaldata/tPACE

For manuscripts utilizing custom algorithms or software that are central to the research but not yet described in published literature, software must be made available to editors and reviewers. We strongly encourage code deposition in a community repository (e.g. GitHub). See the Nature Research guidelines for submitting code & software for further information.

Data

Policy information about availability of data

All manuscripts must include a data availability statement. This statement should provide the following information, where applicable:

- Accession codes, unique identifiers, or web links for publicly available datasets
- A list of figures that have associated raw data
- A description of any restrictions on data availability

The data shown in the manuscript are available upon request from the corresponding author.

Field-specific reporting

Please select the one below that is the best fit for your research. If you are not sure, read the appropriate sections before making your selection.

☒ Life sciences ☐ Behavioural & social sciences ☐ Ecological, evolutionary & environmental sciences

For a reference copy of the document with all sections, see nature.com/documents/nr-reporting-summary-flat.pdf

Life sciences study design

All studies must disclose on these points even when the disclosure is negative.

Sample size	No sample size calculations were done. We used all available retrospective data from our institutional cohort after exclusions.
Data exclusions	Patients were eligible if they were 18 years or older and had at least two encounters at one of 18 medical oncology clinics within the UPHS between January 2nd, 2018 and May 4th, 2020. To ensure that we captured patients who received their primary oncology care in our center and health system, we only included patients who had at least 3 face-to-face visits in an oncology practice for a cancer diagnosis in the year preceding death, and at least two visits in the six months prior to death. Of 44,588 patients who met the criteria above, 41,308 (92.6%) patients who were alive at six months from the index encounter were excluded from this analysis. 3280 deceased patients who had encounters within 6 months of death were eligible for this study.
Replication	This was a retrospective study and we used all available data.
Randomization	This was not relevant for this study as it was not a randomized study.
Blinding	Blinding was not relevant for this retrospective study

Reporting for specific materials, systems and methods

We require information from authors about some types of materials, experimental systems and methods used in many studies. Here, indicate whether each material, system or method listed is relevant to your study. If you are not sure if a list item applies to your research, read the appropriate section before selecting a response.

Materials & experimental systems

n/a	Involved in the study	
☒	☐	Antibodies
☒	☐	Eukaryotic cell lines
☒	☐	Palaeontology and archaeology
☒	☐	Animals and other organisms
☒	☐	Human research participants
☒	☐	Clinical data
☒	☐	Dual use research of concern

Methods

n/a	Involved in the study	
☒	☐	ChIP-seq
☒	☐	Flow cytometry
☒	☐	MRI-based neuroimaging
